# Supplementary material for: Mucosal immune responses and intestinal microbiome associations in wild spotted hyenas (Crocuta crocuta)
Source: Commun Biol. 2025 Jun 13;8:924. doi: 10.1038/s42003-025-08243-0 (PMC12166089; doi:10.1038/s42003-025-08243-0)
Supplement: Supplementary file 1 — Supplementary information [file 42003_2025_8243_MOESM1_ESM.pdf]

Supplements

Mucosal immune responses and intestinal microbiome associations in wild spotted hyenas (*Crocuta crocuta*)

Susana P. V. Soares[1,2*],Victor H. Jarquín-Díaz[3,4§], Miguel M. Veiga[1,2], Stephan Karl[1], Gábor Á. Czirják[2], Alexandra Weyrich[5,6], Sonja Metzger[1], Marion L. East[1], Heribert Hofer[7,8,9], Emanuel Heitlinger[3,4], Sarah Benhaiem[1*], Susana C. M. Ferreira[10*]

[1] Department of Ecological Dynamics, Leibniz Institute for Zoo and Wildlife Research (IZW), Alfred-Kowalke-Strasse 17, 10315 Berlin, Germany
[2] Department of Wildlife Diseases, IZW, Alfred-Kowalke-Strasse 17, 10315 Berlin, Germany
[3] Institute for Biology, Department of Molecular Parasitology, Humboldt University Berlin (HU). Philippstr. 13, Haus 14, 10115, Berlin, Germany
[4] Research Group Ecology and Evolution of Molecular Parasite-Host Interactions, IZW, Alfred-Kowalke-Straße 17, 10315 Berlin, Germany
[5] Department of Evolutionary Genetics, IZW, Alfred-Kowalke-Straße 17, 10315 Berlin, Germany
[6] German Centre for Integrative Biodiversity Research (iDiv), Halle-Jena-Leipzig, Puschstrasse 4, 04103 Leipzig, Germany
[7] Department of Biology, Chemistry, Pharmacy, Freie Universität Berlin, Arnimallee 22, 14195 Berlin, Germany
[8] Department of Veterinary Medicine, Freie Universität Berlin, Oertzenweg 19b 14163 Berlin, Germany
[9] Leibniz Institute for Zoo and Wildlife Research (IZW), Alfred-Kowalke-Strasse 17, 10315 Berlin, Germany
[10] Research Institute of Wildlife Ecology, Department of Interdisciplinary Life Sciences, University of Veterinary Medicine Vienna, Savoyenstrasse 1, 1160 Vienna, Austria

* corresponding author(s)

[§]Current affiliation: Max-Delbrück-Center for Molecular Medicine in the Helmholtz Association (MDC). Robert-Rössle-Str. 10, 13125 Berlin, Germany

Supplementary methods

Primers list
see additional **Supplementary Data 1**

Taxonomic annotation list
see additional **Supplementary Data 2**

Interpretation of relative abundances
The resulting taxa abundances of both single and multi-amplicon are compositional, meaning that their abundances estimates impact each other. Regardless of the inherent biases, e.g. associated with unequal amplification, insufficient coverage of primers, these methods are useful in determining the changes in the relative abundance of taxa within samples [1]. Thus, the interpretation of results must always take into account that the abundance estimates of each taxa within a sample are relative and not absolute. Given this, we refrain from comparing taxa abundances within a sample (e.g. taxon A has higher

abundance than taxon B), and instead focus on the differences between the samples (sample A is similar/dissimilar to sample B, ß-diversity).

Supplementary figures

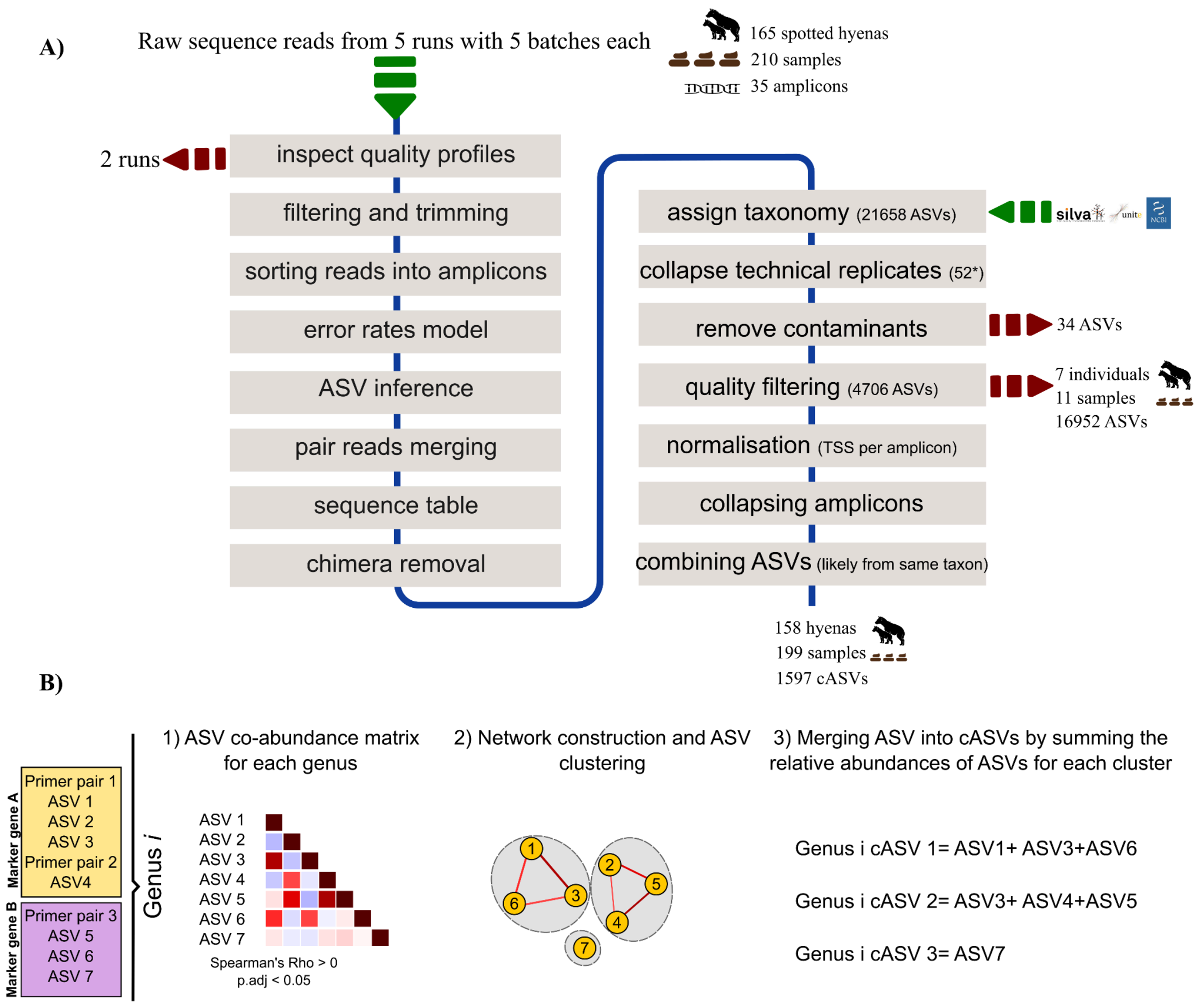

Supplementary Figure 1. A) Overview of the bioinformatics pipeline. In total, 210 samples from 165 spotted hyenas were sequenced on 5 runs with 5 batches each. Upon inspection of the quality profile, 2 runs were excluded. Dada2 pipeline was followed for each amplicon, taxonomic assignment was based on existing dedicated databases, when available, and when not available was based on NCBI sequences. 52 technical replicates were collapsed. 34 ASVs were removed with the decontam approach. Quality filtering consisted of the removal of ASVs that only occurred in one sample (singletons) and samples that had fewer than 100 reads. This resulted in the exclusion of 11 samples from 7 individuals and 16954 ASVs. Normalisation (TSS: total sum scaling) was performed per amplicon before collapsing (collating) all amplicons into one phyloseq object. After combining ASVs likely produced from the same taxon, the final dataset for downstream analysis contained 1597 cASVs from 199 samples of 158 individual spotted hyenas. B) An overview of the combined ASV approach step to deal with ASVs targeting multiple genes or gene regions of the same taxon. A co-abundance matrix based on significant and positive Spearman correlations is created for each genus. Based on this, a network is constructed and ASVs are clustered. ASVs that cluster together are combined into a cASV by summing their relative abundances. Hyena icons were designed by Sonja Metzger.

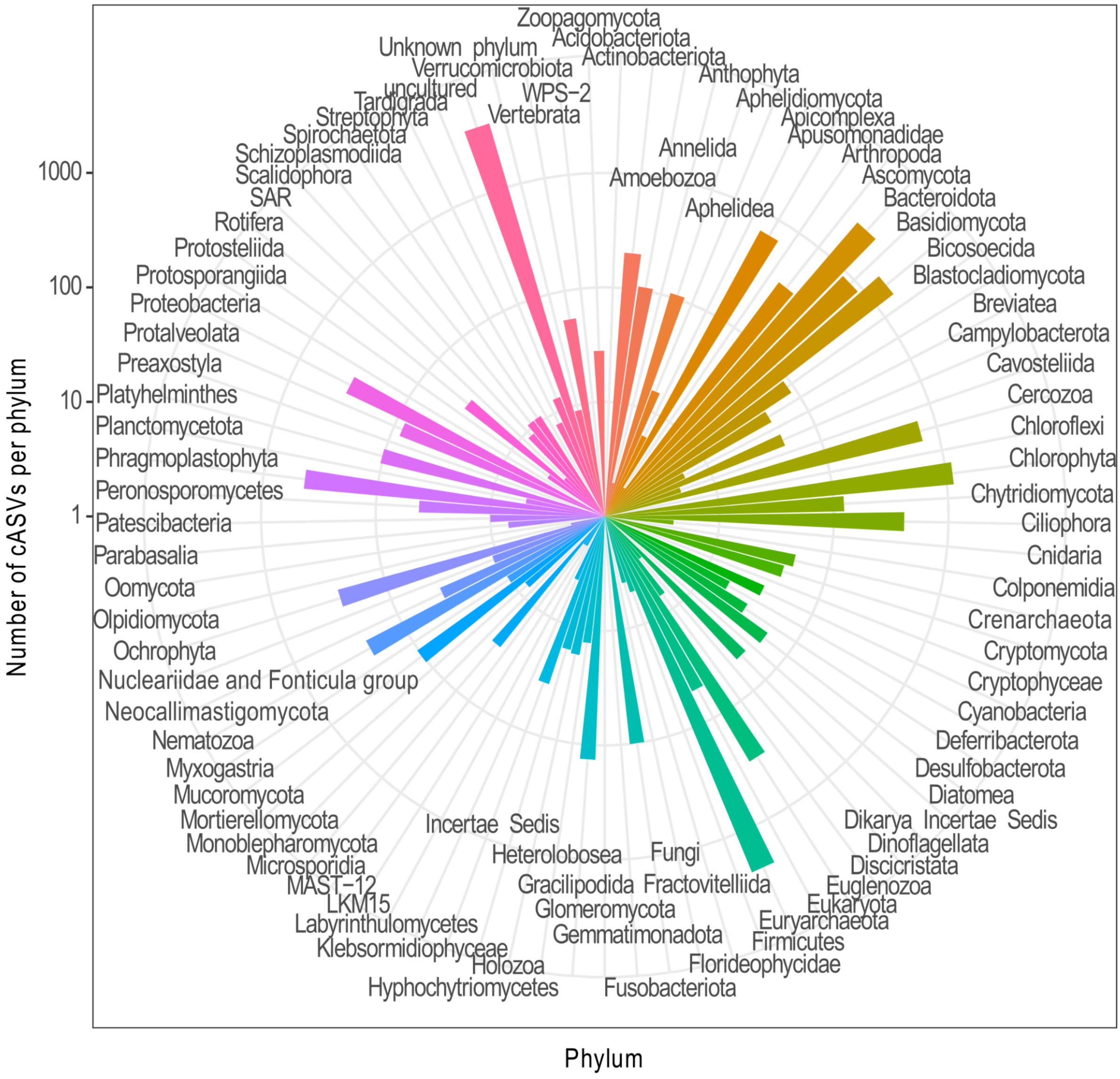

Supplementary Figure 2. Taxonomic annotation of 16869 ASVs present in only 1 sample (singletons) of all 211 samples. All but 5837 ASVs have relative abundances less than 0.005%.

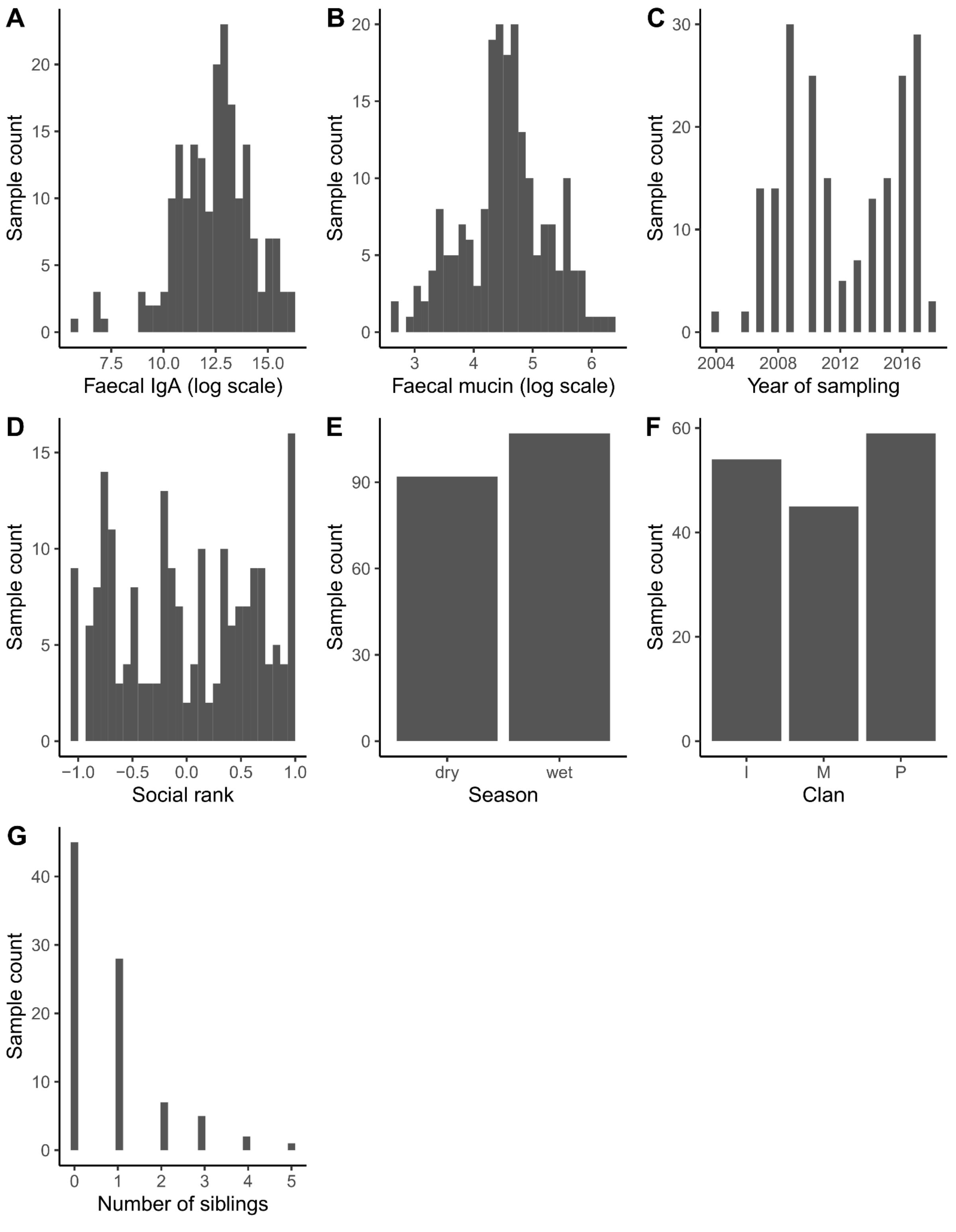

Supplementary Figure 3. Sampling design of the study. A) Histogram of individual measured f-IgA across samples. B) Histogram of individual measured f-mucin across samples. C) Frequency count of the year of sampling across samples. D) Histogram of the individual standardised social rank across samples. E) Frequency count of sampling across seasons. F) Frequency count of sampled individuals belonging to each clan (I, M and P). G) Frequency count of the number of sampled siblings per individual included in this study. Individual count includes 158 individuals and sample count includes 199 samples.

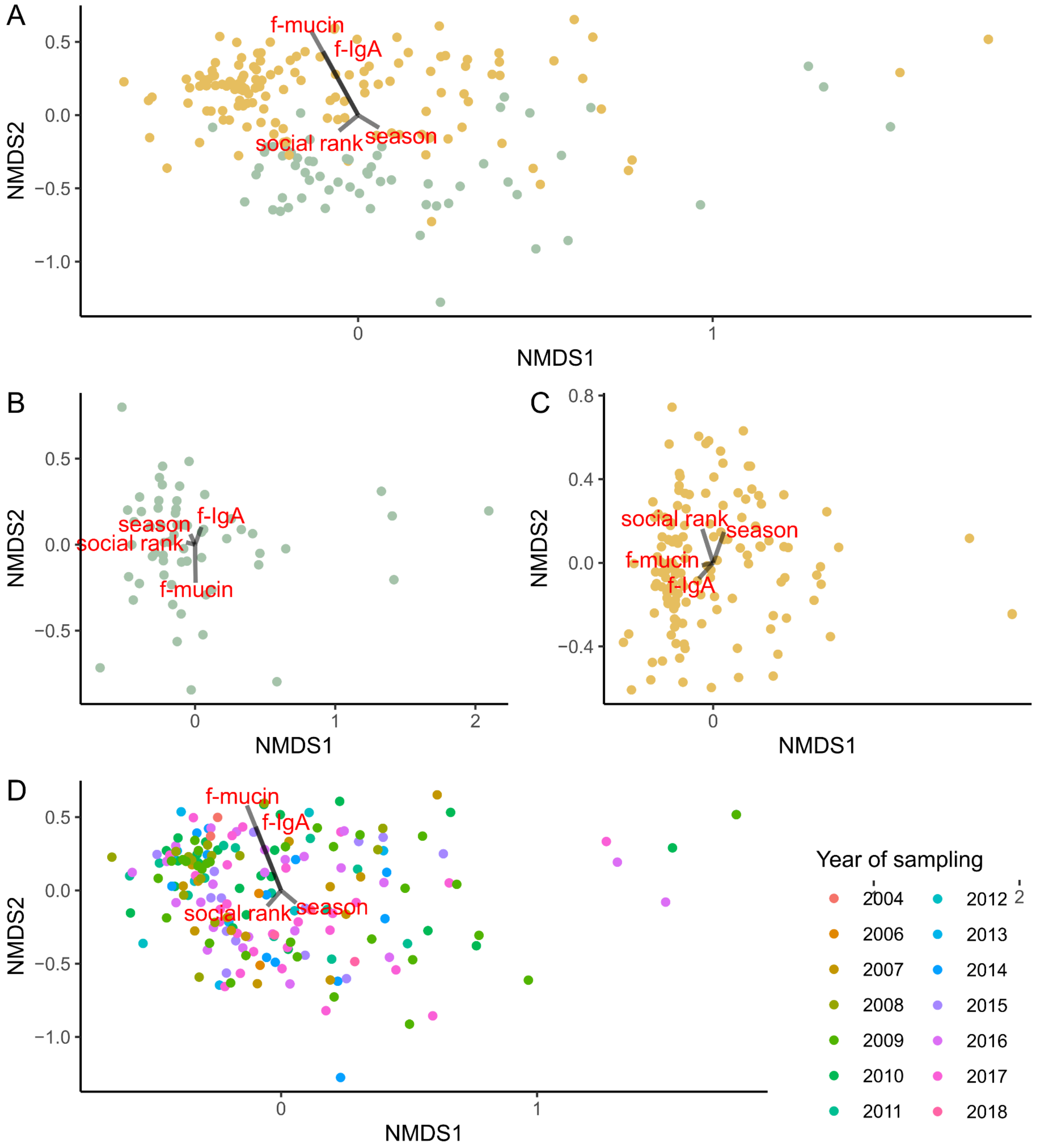

Supplementary Figure 4. Non parametric multidimensional scaling (nMDS) of the overall microbiome composition for A) all samples, n=199, B) adults, n=61 and C) juveniles, n=138. Vectors indicate the direction and magnitude of the correlation between a variable and the ordination axis. C) all samples coloured by year of sampling.

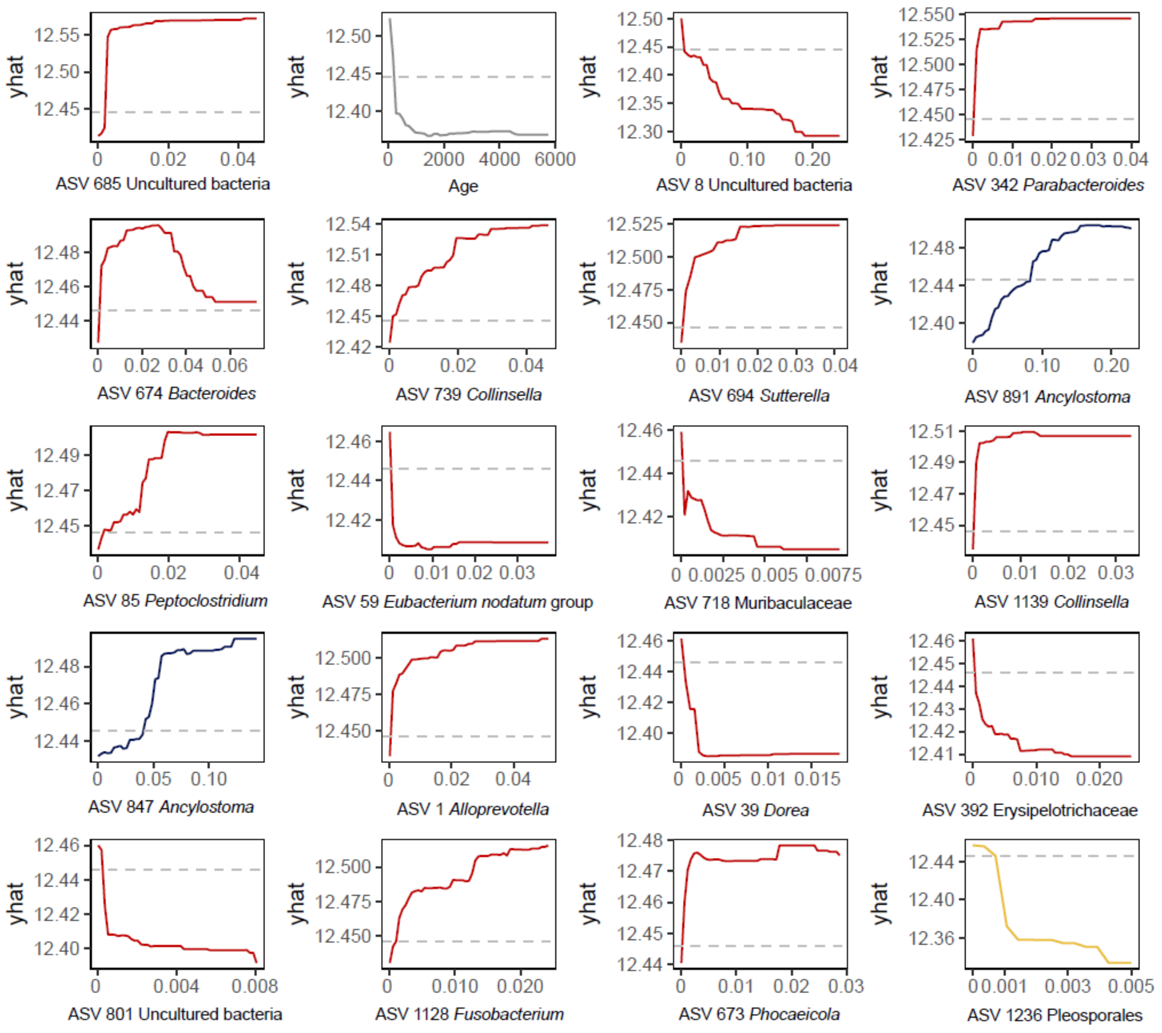

Supplementary Figure 5. Marginal effect size of the 20 most important taxa for prediction of f-IgA.
The relationship between f-IgA and the top 20 cASVs based on partial dependence plots. On the y-axis, yhat is expected f-IgA as a function of the relative abundances of cASVs. Red represents taxa from the bacterial domain; brown, the host DNA and blue, eukaryotic parasite taxa.

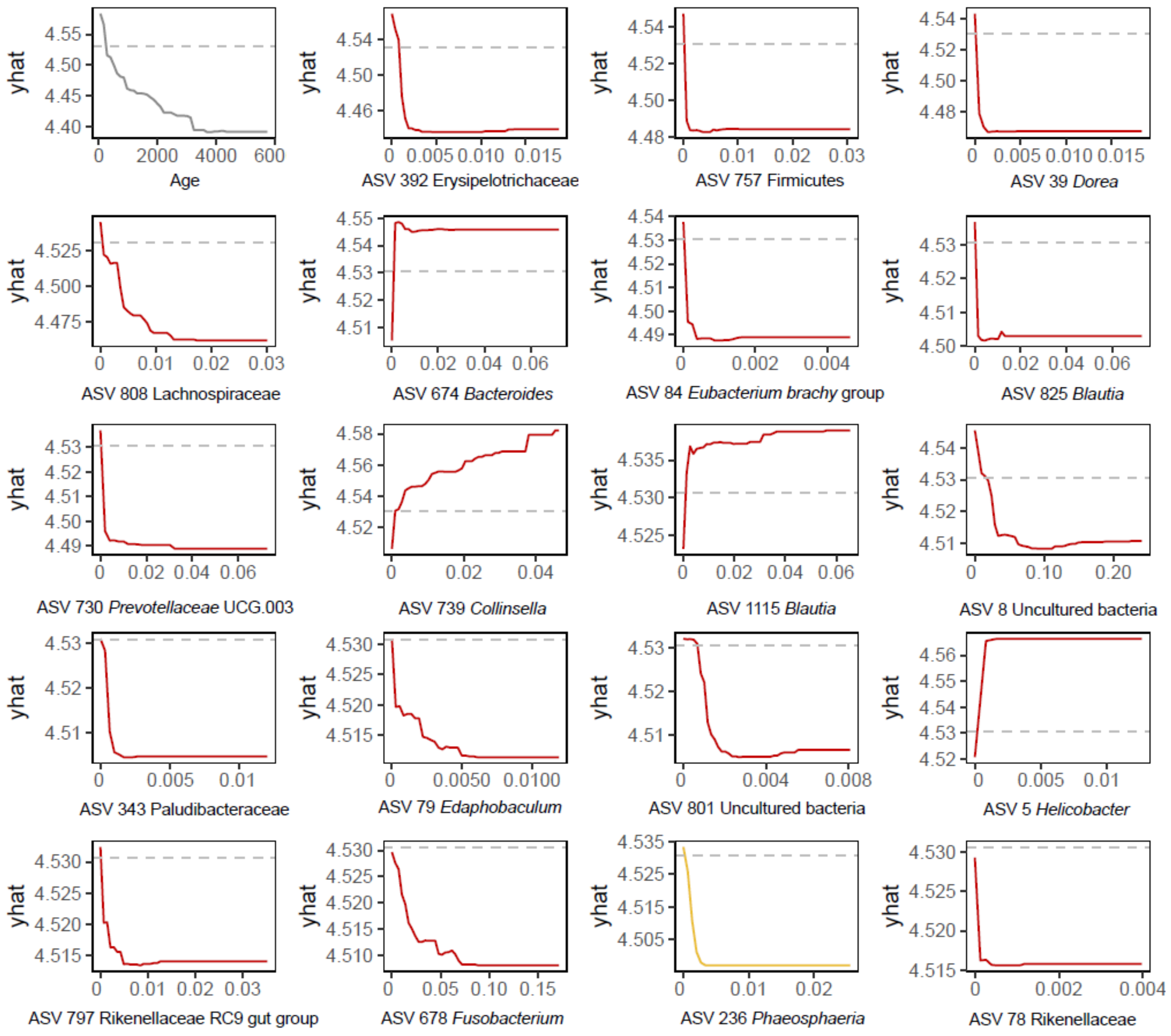

Supplementary Figure 6. Marginal effect size of the 20 most important taxa for prediction of f-mucin.

The relationship between f-mucin and the top 20 cASVs based on partial dependence plots. On the y-axis yhat is expected f-mucin as a function of the relative abundances of cASVs. Red represents taxa from the bacterial domain; brown, the host DNA; blue, eukaryotic parasite taxa; lilac, prey DNA; and yellow, fungi.

Supplementary tables

Supplementary Table 1. Host and ecological factors affect the intestinal community composition similarity (Bray-Curtis, Aitchison and Jaccard similarity distances). Shown are the Bayesian regression multi-membership models testing the effect of each predictor on the overall microbiome similarities (all sequenced ASVs). We show the mean estimates of the posterior distribution for each predictor and the associated 95% credible intervals (95% CI). R-hat values provide information on the chain convergence and are all below 1.01, indicating good convergence. A parameter is considered significant when the 95% CI does not include zero and is shown as coloured. All predictors are expressed in distances between compared pairs, n=19701.

	Bray-Curtis similarity distances (abundance-based)		Aitchison similarity distances (abundance-based)		Jaccard similarity distances (occurrence-based)	
	Estimate	95% CI	Estimate	95% CI	Estimate	95% CI
f-IgA dist	**-0.050**	**-0.062 to -0.038**	**-0.053**	**-0.063 to -0.042**	**-0.049**	**-0.057 to -0.041**
f-mucin dist	**-0.111**	**-0.123 to -0.100**	**-0.058**	**-0.069 to -0.048**	**-0.097**	**-0.105 to -0.089**
Age dist	**-0.149**	**-0.160 to -0.138**	**-0.128**	**-0.139 to -0.117**	**-0.095**	**-0.103 to -0.087**
Social rank dist	0.002	-0.003 to 0.007	0.002	-0.003 to 0.006	0.001	-0.003 to 0.004
Genetic mum [same]	**0.012**	**0.001 to 0.022**	0.010	-2.011 to 0.019	0.003	-0.004 to 0.011
Temporal dist	**-0.013**	**-0.018 to -0.008**	**-0.013**	**-0.018 to -0.008**	**-0.011**	**-0.014 to -0.007**
Clan [same]	**0.003**	**0.0004 to 0.005**	0.002	-0.0002 to 0.004	**0.002**	**0.001 to 0.004**
Season [same]	**0.007**	**0.005 to 0.009**	**0.005**	**0.003 to 0.007**	**0.007**	**0.005 to 0.008**
Age:f-IgA dist	**0.073**	**0.041 to 0.105**	**0.033**	**0.005 to 0.061**	**-0.026**	**-0.047 to -0.004**
Age:f-mucin dist	**0.087**	**0.056 to 0.116**	**0.041**	**0.014 to 0.068**	0.003	-0.018 to 0.023
Batch [same]	**0.032**	**0.029 to 0.035**	**0.028**	**0.025 to 0.030**	**0.013**	**0.010 to 0.015**

Supplementary Table 2. The effects of host-related predictors (immune measures, age) and social and ecological environments on the overall and bacteria, fungi, or parasite-specific intestinal community composition similarity (Bray-Curtis similarity distance) based on Bayesian regression multi-membership models. We show the mean estimates of the posterior distribution for each predictor and the associated 95% credible intervals (CI). All predictors are expressed as distances between compared pairs (n=19701). Effect sizes in bold indicate significant predictors for which both upper and lower 95% credible intervals (CI) are either positive or negative.

	**Bacteria**	**Fungi**	**Parasite**	**Overall**
Predictors	*Estimate (credible interval)*	*Estimate (credible interval)*	*Estimate (credible interval)*	*Estimate (credible interval)*
f-IgA dist	**-0.100**	0.003	**-0.062**	**-0.050**
	**(-0.116 to -0.083)**	(-0.009 to 0.014)	**(-0.087 to -0.037)**	**(-0.062 to -0.038)**
f-mucin dist	**-0.176**	**-0.028**	**-0.078**	**-0.111**
	**(-0.193 to -0.159)**	**(-0.040 to -0.017)**	**(-0.104 to -0.053)**	**(-0.123 to -0.100)**
Age dist	**-0.161**	**0.015**	**-0.112**	**-0.149**
	**(-0.178 to -0.144)**	**(0.004 to 0.028)**	**(-0.138 to -0.087)**	**(-0.160 to -0.138)**
Social rank	0.002	-0.004	0.002	0.002
[same]	(-0.005 to 0.009)	(-0.009 to 0.0005)	(-0.008 to 0.012)	(-0.003 to 0.007)
Genetic mother	0.008	0.009	0.023	**0.012**
[same]	(-0.008 to 0.023)	(-0.001 to 0.020)	(-0.001 to 0.047)	**(0.001 to 0.022)**

Temporal dist	-0.006	**-0.024**	-0.011	**-0.013**
	(-0.014 to 0.002)	**(-0.029 to -0.019)**	(-0.022 to 0.001)	**(-0.018 to -0.008)**
Clan [same]	0.003	0.0003	-0.003	**0.003**
	(-0.0002 to 0.006)	(-0.002 to 0.003)	(-0.008 to 0.002)	**(0.0004 to 0.005)**
Season [same]	**0.007**	**0.012**	0.001	**0.007**
	**(0.004 to 0.010)**	**(0.009 to 0.014)**	(-0.004 to 0.006)	**(0.005 to 0.009)**
Parasite	**0.107**	**0.028**	-	-
similarity	**(0.097 to 0.116)**	**(0.022 to 0.035)**		
Fungi similarity	**0.153**	**-**	**0.135**	**-**
	**(0.133 to 0.173)**		**(0.104 to 0.165)**	
Bacteria	**-**	**0.071**	**0.240**	**-**
similarity		**(0.062 to 0.081)**	**(0.219 to 0.260)**	
Batch [same]	**0.012**	**0.011**	0.006	**0.032**
	**(0.007 to 0.016)**	**(0.007 to 0.014)**	(-0.001 to 0.013)	**(0.029 to 0.035)**
f-IgA dist: Age	-0.043	-0.031	**0.142**	**0.073**
dist	(-0.089 to 0.004)	(-0.062 to 0.0002)	**(0.077 to 0.211)**	**(0.041 to 0.105)**
f-mucin dist: Age	-0.041	**0.046**	**0.145**	**0.087**
dist	(-0.085 to 0.002)	**(0.017 to 0.076)**	**(0.081 – 0.210)**	**(0.056 - 0.116)**

Supplementary Table 3. No detected effect of sex on the intestinal community composition similarity (Bray-Curtis) in 138 samples from 122 juvenile spotted hyenas. Shown are the Bayesian regression multi-membership models testing the effect of each predictor on the overall microbiome similarities (all sequenced ASVs) and parasite, fungi and bacteria component similarities. We show the mean estimates of the posterior distribution for each predictor and the associated 95% credible intervals (95% CI). R-hat values provide information on the chain convergence and do not exceed 1.01, indicating good convergence. All predictors are expressed in distances between compared pairs, n=9453.

	Overall		Parasite		Fungi		Bacteria	
	Estimate	95% CI	Estimate	95% CI	Estimate	95% CI	Estimate	95% CI
Sex [MM]	0.018	-0.024 to 0.061	0.048	-0.070 to 0.167	0.020	-0.014 to 0.055	0.017	-0.018 to 0.054
Sex [FM]	0.010	-0.011 to 0.032	0.023	-0.036 to 0.082	0.011	-0.006 to 0.028	0.009	-0.009 to 0.028
f-IgA dist	**-0.040**	**-0.049 to -0.030**	**-0.027**	**-0.049 to -0.005**	-0.001	-0.010 to 0.009	**-0.064**	**-0.078 to -0.051**
f-mucin dist	**-0.042**	**-0.053 to -0.030**	**-0.047**	**-0.073 to -0.021**	**-0.039**	**-0.051 to -0.028**	**-0.064**	**-0.080 to -0.048**
Age dist	**-0.159**	**-0.176 to -0.143**	**-0.088**	**-0.125 to -0.052**	-0.009	-0.026 to 0.007	**-0.378**	**-0.401 to -0.355**
Temporal dist	**-0.031**	**-0.039 to -0.023**	**-0.028**	**-0.045 to -0.011**	**-0.033**	**-0.041 to -0.025**	**-0.023**	**-0.035 to -0.012**
Clan [same]	**0.004**	**0.001 to 0.008**	0.0009	-0.006 to 0.008	0.001	-0.002 to 0.004	**0.005**	**0.0003 to 0.009**
Season [same]	**0.003**	**0.0004 to 0.006**	**0.008**	**0.002 to 0.015**	**0.008**	**0.005 to 0.012**	**0.004**	**0.0003 to 0.008**
Batch [same]	**0.035**	**0.031 to 0.039**	**0.018**	**0.009 to 0.027**	**0.008**	**0.004 to 0.012**	**0.015**	**0.009 to 0.021**

Supplementary references

1. Nearing, J. T., Comeau, A. M. & Langille, M. G. I. Identifying biases and their potential solutions in human microbiome studies. *Microbiome* **9**, 113 (2021).
