# Supplementary material for: Mucosal immune responses and intestinal microbiome associations in wild spotted hyenas (Crocuta crocuta)
Source: Commun Biol. 2025 Jun 13;8:924. doi: 10.1038/s42003-025-08243-0 (PMC12166089; doi:10.1038/s42003-025-08243-0)
Supplement: Supplementary file 2 — Description of Additional Supplementary Materials [file 42003_2025_8243_MOESM2_ESM.pdf]

Description of Additional Supplementary Files

File name: Supplementary Data 1

Description: Primers list.

File name: Supplementary Data 2

Description: Taxonomic annotation list.
