# Supplementary material for: Mucosal immune responses and intestinal microbiome associations in wild spotted hyenas (Crocuta crocuta)
Source: Commun Biol. 2025 Jun 13;8:924. doi: 10.1038/s42003-025-08243-0 (PMC12166089; doi:10.1038/s42003-025-08243-0)
Supplement: Supplementary file 5 — Reporting Summary [file 42003_2025_8243_MOESM5_ESM.pdf]

nature portfolio

Corresponding author(s): Susana P.V. Soares

Last updated by author(s): May 16, 2025

Reporting Summary

Nature Portfolio wishes to improve the reproducibility of the work that we publish. This form provides structure for consistency and transparency in reporting. For further information on Nature Portfolio policies, see our Editorial Policies and the Editorial Policy Checklist.

Statistics

For all statistical analyses, confirm that the following items are present in the figure legend, table legend, main text, or Methods section.

n/a	Confirmed	
☐	☒	The exact sample size (*n*) for each experimental group/condition, given as a discrete number and unit of measurement
☐	☒	A statement on whether measurements were taken from distinct samples or whether the same sample was measured repeatedly
☐	☒	The statistical test(s) used AND whether they are one- or two-sided<br>*Only common tests should be described solely by name; describe more complex techniques in the Methods section.*
☐	☒	A description of all covariates tested
☐	☒	A description of any assumptions or corrections, such as tests of normality and adjustment for multiple comparisons
☐	☒	A full description of the statistical parameters including central tendency (e.g. means) or other basic estimates (e.g. regression coefficient) AND variation (e.g. standard deviation) or associated estimates of uncertainty (e.g. confidence intervals)
☒	☐	For null hypothesis testing, the test statistic (e.g. *F*, *t*, *r*) with confidence intervals, effect sizes, degrees of freedom and *P* value noted<br>*Give P values as exact values whenever suitable.*
☐	☒	For Bayesian analysis, information on the choice of priors and Markov chain Monte Carlo settings
☒	☐	For hierarchical and complex designs, identification of the appropriate level for tests and full reporting of outcomes
☐	☒	Estimates of effect sizes (e.g. Cohen's *d*, Pearson's *r*), indicating how they were calculated

Our web collection on statistics for biologists contains articles on many of the points above.

Software and code

Policy information about availability of computer code

Data collection	We collected faeces, extracted genetic material and performed next-generation sequencing within Illumina MiSeq platform. Data is available at BioProject PRJNA1134446 in the NCBI Short Read Archive
Data analysis	Analysis was performed with R 4.4.0 and packages. Code and outputs available at https://github.com/ferreira-scm/Microbiome_Hyena.git or through the Zenodo Digital Repository (doi:10.5281/zenodo.15283097).

For manuscripts utilizing custom algorithms or software that are central to the research but not yet described in published literature, software must be made available to editors and reviewers. We strongly encourage code deposition in a community repository (e.g. GitHub). See the Nature Portfolio guidelines for submitting code & software for further information.

Data

Policy information about availability of data

All manuscripts must include a data availability statement. This statement should provide the following information, where applicable:

- Accession codes, unique identifiers, or web links for publicly available datasets
- A description of any restrictions on data availability
- For clinical datasets or third party data, please ensure that the statement adheres to our policy

The statistical analysis and data are available at BioProject PRJNA1134446 in NCBI Short Read Archive and at https://github.com/ferreira-scm/

Microbiome_Hyena.git or through the Zenodo Digital Repository (doi:10.5281/zenodo.15283097).

Research involving human participants, their data, or biological material

Policy information about studies with human participants or human data. See also policy information about sex, gender (identity/presentation), and sexual orientation and race, ethnicity and racism.

Reporting on sex and gender	NA
Reporting on race, ethnicity, or other socially relevant groupings	NA
Population characteristics	NA
Recruitment	NA
Ethics oversight	NA

Note that full information on the approval of the study protocol must also be provided in the manuscript.

Field-specific reporting

Please select the one below that is the best fit for your research. If you are not sure, read the appropriate sections before making your selection.

☐ Life sciences ☐ Behavioural & social sciences ☒ Ecological, evolutionary & environmental sciences

For a reference copy of the document with all sections, see nature.com/documents/nr-reporting-summary-flat.pdf

Ecological, evolutionary & environmental sciences study design

All studies must disclose on these points even when the disclosure is negative.

Study description	We investigated associations between the intestinal microbiome and mucosal immune measures while controlling for host, social and ecological factors in 199 samples of 158 wild spotted hyenas (Crocuta crocuta) in the Serengeti National Park, Tanzania. We profiled the microbiome composition using a multi-amplicon approach and measured faecal immunoglobulin A and mucin. Probabilistic models indicated that both immune measures predicted microbiome similarity among individuals in an age-dependent manner. These associations were the strongest within bacteria, intermediate within parasites, and weakest within fungi communities. Machine learning models accurately predicted both immune measures and identified the taxa driving these associations: symbiotic bacteria reported in humans and laboratory mice, unclassified bacteria, parasitic hookworms and fungi. These findings improve our understanding of the gut microbiome, its drivers, and interactions in wild populations under natural selection.
Research sample	We non-invasively collected 199 faecal samples of 158 individually known wild spotted hyenas from the Serengeti National Park, Tanzania.
Sampling strategy	During each field session we routinely scored the presence of all clan members present near the communal den(s). Non-invasive faecal samples were routinely, opportunistically and immediately collected after defecation. We used the same research vehicle to which animals are habituated and were careful not to be seen by animals when collecting the samples. We did so by positioning the vehicle very close to the faeces, to both guarantee the safety of the researchers and minimise disturbance to the animals. Samples were collected in individual labelled bags and refrigerated in cool boxes with frozen ice packs in the field until transport to the field station (no more than 3-4 hours later). At the field station, samples were mechanically mixed and aliquots stored at -10°C until their transport to storage at - 80°C at the IZW. Aliquots for DNA extraction were stored in RNAlater (Sigma-Aldrich, St Louis, MO, USA).
Data collection	Individuals are recognised based on their unique spot patterns and other features, such as scars and ear notches. We limited our focus to cubs of both sexes and adult females as most adult males tend to disperse upon reaching adulthood, making it challenging to monitor them throughout their entire lifespan.<br>Cubs were aged to an accuracy of ±7days based on their behaviour, movement coordination, size and pelage when seen for the first time. By the age of approximately three months, sex was determined by the shape of the external genitalia, particularly the dimorphic glans morphology of the erect phallus. Maternal identity was determined based on nursing observations at the communal den(s) and was further confirmed by DNA microsatellite loci analysis . The social rank of adult females in their clans was determined based on the observation of submissive acts in dyadic interactions recorded ad libitum and during focal observations . For each clan, we used the outcome of these dyadic interactions to construct an adult female linear dominance hierarchy that was updated daily after demographic changes (recruitment or deaths of adult females) and socially mediated changes in rank (coups).
Timing and spatial scale	Serengeti National Park (Tanzania) from 2004-2018, in the context of a long-term research project initiated in 1987.We collected data and faecal samples oportunistically and non-invasively from individually known spotted hyenas.
Data exclusions	n/a
Reproducibility	Code, database and protocols are accessible.

Randomization	n/a
Blinding	For unbiased treatment: lab analysis and data analysis were conducted blindly from one another.

Did the study involve field work? ☒ Yes ☐ No

Field work, collection and transport

Field conditions	Non-invasive monitoring and sampling in the Serengeti National Park, Tanzania.
Location	Serengeti National Park (Tanzania) between 2004-2018.
Access & import/export	For fieldwork, we were granted research permits from the Tanzania Commission for Science and Technology (COSTECH) and permission from the Tanzanian National Parks Authority (TANAPA) and Tanzanian Wildlife Research Institute (TAWIRI). For the export of biological samples samples from Tanzania to Germany, we received permission from TAWIRI, the CITES office of IUCN in Tanzania and the Tanzanian officer responsible for sample export in compliance with the Nagoya Convention.
Disturbance	Minimal

Reporting for specific materials, systems and methods

We require information from authors about some types of materials, experimental systems and methods used in many studies. Here, indicate whether each material, system or method listed is relevant to your study. If you are not sure if a list item applies to your research, read the appropriate section before selecting a response.

Materials & experimental systems

n/a	Involved in the study	
☐	☒	Antibodies
☒	☐	Eukaryotic cell lines
☒	☐	Palaeontology and archaeology
☐	☒	Animals and other organisms
☒	☐	Clinical data
☒	☐	Dual use research of concern
☒	☐	Plants

Methods

n/a	Involved in the study	
☒	☐	ChIP-seq
☒	☐	Flow cytometry
☒	☐	MRI-based neuroimaging

Antibodies

Antibodies used	Anti-cat IgA (Lot.A10, Novusbio, Abingdon, UK) as capture antibody, and conjugated anti-cat IgA (Lot.P18, Novusbio, Abingdon, UK) as detection antibody.
Validation	Ferreira et al 2021 - doi:10.1002/ece3.7602

Animals and other research organisms

Policy information about studies involving animals; ARRIVE guidelines recommended for reporting animal research, and Sex and Gender in Research

Laboratory animals	n/a
Wild animals	199 non-invasively collected faecal samples of 158 individually known wild spotted hyenas.
Reporting on sex	All adults were female (61 samples from 58 individuals) because they are philopatric and juveniles were of both sexes (138 samples from 41 males and 81 females). Sex was determined from the age of 3 months based on the shape of the external genitalia, particularly the dimorphic glans morphology of the erect phallus.
Field-collected samples	All samples were collected routinelly, opportunistically and non-invasively during the field sessions. Samples were collected in individual labelled bags and refrigerated in cool boxes with frozen ice packs in the field until transport to the field station (no more than 3-4 hours later). At the field station, they were mechanically mixed and aliquots stored at -10°C until their transport to storage at - 80°C at the IZW. Aliquots for DNA extraction were stored in RNAlater (Sigma-Aldrich, St Louis, MO, USA).
Ethics oversight	For fieldwork, we were granted research permits from the Tanzania Commission for Science and Technology (COSTECH) and permission from the Tanzanian National Parks Authority (TANAPA) and Tanzanian Wildlife Research Institute (TAWIRI). All procedures

were performed in accordance with the Leibniz Institute for Zoo and Wildlife Research Ethics Committee on Animal Welfare (permit number: 2017-11-02).

Note that full information on the approval of the study protocol must also be provided in the manuscript.

Plants

Seed stocks

NA

Novel plant genotypes

NA

Authentication

NA
